# Synthesis of Nitric Oxide Donors Derived from Piloty’s Acid and Study of Their Effects on Dopamine Secretion from PC12 Cells

**DOI:** 10.3390/ph10030074

**Published:** 2017-09-05

**Authors:** Daniele Sanna, Gaia Rocchitta, Maria Serra, Marcello Abbondio, Pier Andrea Serra, Rossana Migheli, Lidia De Luca, Eugenio Garribba, Andrea Porcheddu

**Affiliations:** 1 Istituto CNR di Chimica Biomolecolare, Trav. La Crucca 3, 07040 Sassari, Italy; Daniele.Sanna@icb.cnr.it (D.S.); Maria.Serra@icb.cnr.it (M.S.); 2 Department of Clinical and Experimental Medicine, Medical School, University of Sassari, viale San Pietro 43/b, 07100 Sassari, Italy; grocchitta@uniss.it (G.R.); rmigheli@uniss.it (R.M.); 3 Department of Biomedical Sciences, University of Sassari, 07100 Sassari, Italy; mabbondio@uniss.it; 4 Department of Chemistry and Pharmacy, University of Sassari, via Vienna 2, 07100 Sassari, Italy; ldeluca@uniss.it; 5 Department of Chemical and Geological Sciences, University of Cagliari, S.S. 554, bivio per Sestu, 09042 Monserrato, Italy

**Keywords:** Piloty’s acid, nitric oxide, Parkinson’s disease, in vitro microdialysis

## Abstract

This study investigated the mechanisms and kinetics of nitric oxide (NO) generation by derivatives of Piloty’s acid (NO-donors) under physiological conditions. In order to qualitatively and quantitatively measure NO release, electron paramagnetic resonance (EPR) was carried out with NO spin trapping. In addition, voltammetric techniques, including cyclic voltammetry and constant potential amperometry, were used to confirm NO release from Piloty’s acid and its derivatives. The resulting data showed that Piloty’s acid derivatives are able to release NO under physiological conditions. In particular, electron-withdrawing substituents favoured NO generation, while electron-donor groups reduced NO generation. In vitro microdialysis, performed on PC12 cell cultures, was used to evaluate the dynamical secretion of dopamine induced by the Piloty’s acid derivatives. Although all the studied molecules were able to induce DA secretion from PC12, only those with a slow release of NO have not determined an autoxidation of DA itself. These results confirm that the time-course of NO-donors decomposition and the amount of NO released play a key role in dopamine secretion and auto-oxidation. This information could drive the synthesis or the selection of compounds to use as potential drugs for the therapy of Parkinson’s disease (PD).

## 1. Introduction

Almost 20 years ago, the first reports concerning the endogenous generation of nitric oxide (NO) in mammalian systems appeared [1,2,3]. Since these initial reports, the physiology, biochemistry and pharmacology of NO and related compounds have received growing attention [4]. NO is a valuable lead compound of a family of well-established signalling molecules. It is bio-synthesized via oxidation of the amino group of L-arginine, yielding NO and citrulline, the reaction is catalysed by nitric oxide synthases. Three NO synthases (NOS) are known: endothelial (eNOS), neuronal (nNOS), and inducible (iNOS) NOS [5]. Both nNOS and eNOS are constitutive isoforms, which generate low concentrations of NO. In contrast, iNOS can be induced by various cytokines or lipopolysaccharides and produces large amounts of NO over long time periods, often resulting in harmful effects. In the higher animals, NO induces vasodilatation, neurotransmission and vessel homeostasis and is involved in the regulation of respiration, digestion, sexual function, memory, sleep and cardiovascular function. Furthermore, NO has also been correlated with several diseases, such as stroke, diabetes, cancer and cardiovascular disease [6]. In recent years, it has been demonstrated that NO can be a fundamental actor in the central nervous system (CNS), particularly in intracellular signalling [7] and tissue damage/protection [8,9]. Regulation of striatal dopamine (DA) neurotransmission is one of the most important roles for endogenous NO in the CNS [10]. Several animal model studies have demonstrated that NO-donors can increase extracellular levels of DA [11,12,13]. It has been previously shown that NO-mediated DA release could depend on the source of NO, whether neuronal, glial, extracellular, exogenous [8,9], and the timing of NO generation [14].

From a strictly chemical point of view, NO is a simple hydrophobic gaseous molecule that is highly diffusible and reactive. The following forms are important for biological action. NO free radical is a highly versatile species with a low ionization potential that allows it to be easily oxidized to nitrosonium cation (NO^+^), and quickly reduced to nitroxyl anion (NO^−^) [15]. Among them, NO is by far the most stable and the one which has aroused tremendous biological interest and extensive studies. It is known from the literature [8] that the nitrosonium ion can give rise to two main types of compound. The first is ionic salts such as NO^+^BF_4_^−^, while good example of the second type of compound found are the nitroxyl halides. It deserves to be emphasized that both species are quickly hydrolyzed forming nitrous acid (HNO_2_) and NO^+^ can be detected in aqueous solution only at very low pH. Numerous reagents may also reduce NO radical and the degree of reduction may be more or less effective with a wide range of products (N_2_O, N_2_O_2_^2−^, NO^−^, etc.). Simple one-electron reduction of NO affords the nitroxyl anion, NO^−^. The average reducing potential of this redox process is about +0.25 V. The endogenous environment in which NO is generated may also regulate its biological actions [16,17,18,19]. Despite a true understanding of the physiological role played by NO in vivo is an important milestone, this task is far from being trivial [16,17]. Nowadays, while the chemistry of NO is well-established, the exact biochemistry of NO is somewhat blurred [18]. To make matter worse, real-time quantitative measurements of NO from NOS in biological media are analytically challenging and time consuming as result of the labile nature of NO [20]. Even the choice of the most proper analytical method for determining NO in biological matrices is not entirely off-set [21]. In this respect, the application and comparison of different analytical techniques are needed to gain better insights into the physiology and pathology of NO [22,23,24,25,26].

**Figure 1:**
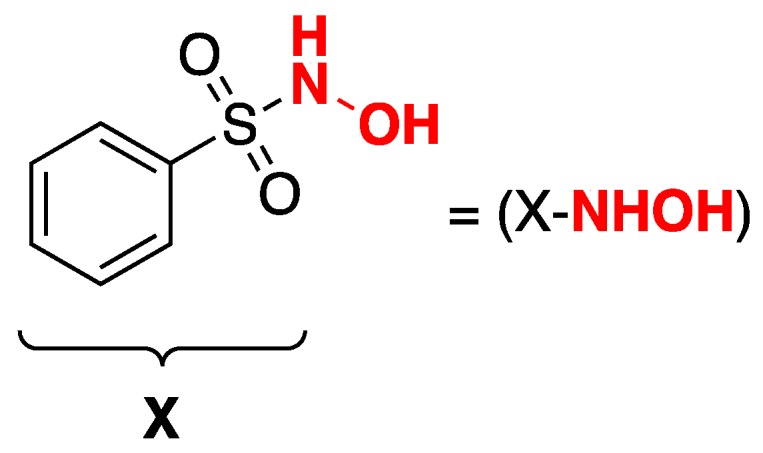
Piloty’s acid (PI) molecular structure.

Direct administration of NO to mimic nNOS-mediated NO production for in vitro studies is not possible due to its instability in aqueous media and/or when oxygen is present. Therefore, chemical reagents that can continuously release NO under physiological conditions (NO-donors) are increasingly important. Generally, NO-donors have different structures, which release NO at different rates and through three different kinds of mechanisms. The first route is the one that releases NO spontaneously via a thermal or photochemical self-decomposition process. In the second route NO is released by a chemical reaction with acids, alkalis, metals and thiols. The third route is an enzymatic oxidation process. Some NO donors can generate NO by following different pathways. The most widely used NO-donors include sodium nitroprusside (SNP) [27], 3-morpholinosydnonimine (SIN-1) [28], *S*-nitroso-*N*-acetylpenicillamine (SNAP) [27] and sodium trioxodinitrate (Angeli’s salt) [29] and glycerin trinitrate (GTN) [27]. First reported in 1896 by the German chemist, Oskar Piloty, benzenesulfohydroxamic acid, commonly referred to as Piloty’s acid (PI), has been found to release NO under certain condition [30]. From a structural point of view, Piloty’s acid is an *N*-substituted hydroxylamine derivative (PhSO_2_NHOH) bearing a good leaving group (a benzenesulfinate unit) on nitrogen moiety (HONH-X, Figure 1). Depending on the nature of the X group and experimental conditions (pH value, temperature, presence of oxidizing or reducing agents) Piloty’s acid can release HNO, NO or both [31,32,33]. Generally, nitroxyl (HNO) is generated from Piloty’s acid by spontaneous decomposition in strongly basic (pH 13) solution and under anaerobic conditions [34].

**Scheme 1:**
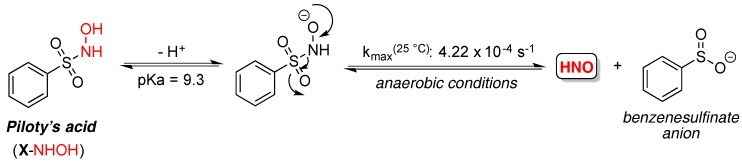
Mechanism proposed for the spontaneous decomposition of Piloty’s acid (PI) in strongly basic (pH 13) solution and under anaerobic conditions.

Briefly, the initial deprotonation at the nitrogen atom is followed by the heterolytic S-N bond cleavage to afford HNO and benzenesulfinate anion (Scheme 1). Since p*K_a_* of HNO is approximately 11.4, it is reasonable to believe that nitroxyl anion (NO^−^) will be the predominant species capables of existing under these conditions [35].

**Scheme 2:**
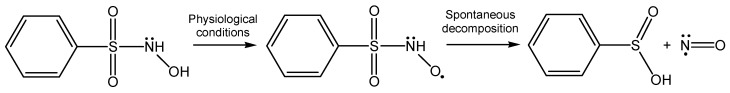
Mechanism proposed for the decomposition of Piloty’s acid (PI) under physiological conditions.

At pH 13, Piloty’s acid decomposes with the first-order rate constant of 1.8 × 10^−3^ s^−1^ at 37 °C [36], whereas the rate of HNO release in aqueous solutions at neutral pH significantly decreases (*t*^1/2^ about 80 h, at 25 °C) making its use most effective above pH 8.0 [31,37] The results revealed that the half-life of Piloty’s acid dramatically decreases with increasing pH values: 561 min at pH 8, 90 min at pH 9, and 33 min at pH 10 [38,39]. The exclusion of oxygen is important since its presence causes oxidative decomposition of Piloty’s acid (non-HNO pathway), which ultimately release nitric oxide (NO) rather than HNO and becomes an NO donor [40]. In 1994, Grzesiok et al. observed that under physiological conditions, Piloty’s acid is oxidized to a radical that spontaneously decomposes to give NO along with the sulfinate leaving group (Scheme 2) [41]. This may explain the vasodilator and antiplatelet activity of PI [42], as well as its potential anti-inflammatory activity. Under physiological conditions, however Piloty’s acid derivatives can decompose though more than one route and it is very difficult entirely rule out the presence/absence of NO or HNO [43].

**Figure 2:**
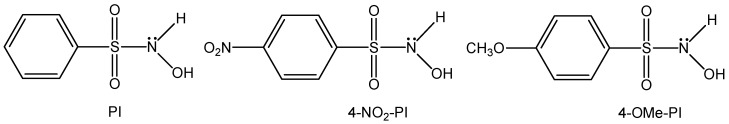
Structure of *N*-hydroxy-benzenesulfonamide (PI), *N*-hydroxy-4-nitrobenzenesulfonamide (4-NO_2_-PI) and *N*-hydroxy-4-methoxybenzenesulfonamide (4-OMe-PI).

This can rise to critical confusion in some experiments since HNO and NO shares similar effects, although they follow different cellular signalling pathways. Recently, in a very interesting paper, Miyata et al., prepared a series of Piloty’s acids derivatives and tested their HNO-releasing activity under physiological conditions by GC-MS detection of nitrous oxide (N_2_O), the dimerization product of HNO (derived from the spontaneously dimerization of HNO) [44]. The results of this study confirmed the importance of initial Toscano’s findings [35,37] and established that the presence of electron-withdrawing groups at the *ortho*-position together with appropriate bulky alkyl residues at the *o*-position promote HNO release at physiological pH. Among all Piloty’s acid derivatives evaluated, *N*-Hydroxy-2-nitro-benzenesulfonamide and 2-bromo-*N*-hydroxybenzenesulfonamide have proven the most efficient HNO donors at pH = 7 releasing HNO in aqueous solution without co-production of NO [33]. A recent similar study conducted by Doctorovich et al., (2013) highlighted that, contrary to PI, *N*-hydroxybenzenesulfonamides with electron-attracting or withdrawing substituents in para-position on the aromatic ring behave preferentially as HNO donors operating at physiological pH and under strictly anaerobic condition [45]. On the contrary, a study published in the same year by Miyata et al., pointed out that Piloty’s acid derivatives having electron-withdrawing groups on the aromatic ring at the para-position released only a negligible amount of HNO by decomposition under physiological oxidative conditions [44]. Therefore, some of these molecules might be ideal candidates for therapeutic treatments responsive to NO and more in-depth investigations were necessary to further explore those knowledge gaps [46,47,48,49]. In this study, we were intrigued by the possibility of using some derivatives of the commercial available Piloty’s acid (PI) as potential source of solely NO. In this regard, *N*-hydroxy-4-nitrobenzenesulfonamide (4-NO_2_-PI) and *N*-hydroxy-4-methoxybenzene sulfonamide (4-OMe-PI) have been synthesized and investigated in depth to exploit them as NO-donors under oxidative stress (Figure 2).

Thus, to contribute to the elucidation of the decomposition mechanism of some select Piloty’s molecular structures [50], the generation of NO species from these derivatives was investigated using electron paramagnetic resonance (EPR) spectroscopy in association with cyclic voltammetry (CV) and constant-potential amperometry (CPA). As NO is a paramagnetic free radical, EPR spectroscopy is the most appropriate tool for its direct detection. The main advantage of EPR with respect to other techniques is that it only detects paramagnetic molecules, and so NO can be measured without the interference of other diamagnetic species; the disadvantage is that the release of HNO or other derivatives cannot be revealed [51]. Because of the high reactivity of NO, spin-trapping techniques have been developed to form relatively stable free radicals [52]. Spin trapping using Fe^2+^ complexes with dithiocarbamates has been demonstrated an excellent procedure to reveal selectively NO [53]. Since the HNO eventually produced can be oxidized to NO and this process can be favoured by the coordination with the spin trapper, EPR alone may not be able to clearly discriminate NO formed directly by the decomposition of PI from that derived by the oxidation of HNO. Fortunately, a number of studies reported in the literature on the reactivity of ^1^HNO with molecular oxygen seems to exclude the possibility that the generation of NO species may takes place by this route since this reaction is rather slow (k = 10^3^ M^−1^ s^−1^) [52,53], mainly due to their different spin states. The combined application of EPR and other techniques can help the interpretation of the experimental data. The electrochemical sensors used in this study are able to detect NO with a high grade of sensitivity and selectivity. In fact, while no scientific papers are present in literature about the HNO interference in NO electrochemical detection assessed by poly-OPD/nafion amperometric sensors, Suárez and coworkers developed specific sensors for the detection of HNO [54]. The mentioned authors showed that only in the presence of a cobalt porphyrin it is possible to perform the amperometric detection of HNO.

In this work, we have studied, in detail, the time profile of NO generation and the mechanism by which these two molecules generate NO under physiological conditions (pH 7.4, 37 °C). The results for 4-NO_2_-PI and 4-OMe-PI were consistent with those of PI and another benchmark NO-donor (*S*-nitroso-*N*-acetylpenicillamine, SNAP) [27]. Finally, an in vitro study of the neurochemical effects of the new PI derivatives was also carried out. In particular, the capability of PI derivatives to induce the dynamical secretion of dopamine on PC12 cell cultures was evaluated.

## 2. Results

### 2.1. Chemicals

Exploiting the propensity of sulfohydroxamic acids to release NO [55,56,57,58,59], we have designed an environmentally friendly, multigram scale synthesis of Piloty’s acid analogues through the reaction of commercially available sulfonyl chlorides with hydroxylamine hydrochloride in a basic medium.

**Scheme 3:**
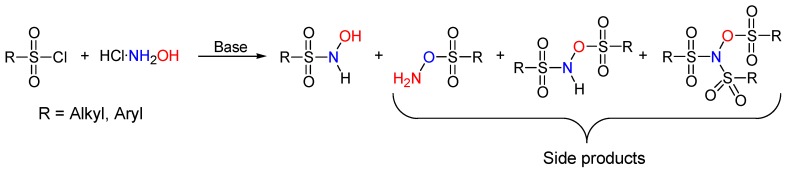
Direct sulfonylation of hydroxylamine under basic condition.

A careful survey of the literature identified several factors that limit the scope of classical sulfonylation reactions with NH_2_OH [31,58]. In fact, the sulfonylation of weakly nucleophilic amines, such as hydroxylamine, is difficult and sulfohydroxamic acids are often obtained in low yields after several elaborate and tedious purification steps [60,61]. On the other hand, the use of activated sulfohydroxamic acids with better leaving groups than chlorine does not improve the yield due to the high reactivity of sulfonyl chlorides. Additionally, direct sulfonylation of hydroxylamine also gives rise to a mixture of *N*- and *O*-sulfonylated side products (Scheme 3) [62,63,64,65,66,67].

In an attempt to address these problems, we have exploited the utility of MgO as a base for the coupling of less reactive hydroxylamines with a set of commercially available sulfonyl chlorides. In the presence of MgO, the sulfonyl chloride group selectively reacts with the more nucleophilic amino group (the *N* in NH_2_OH is a better electron donor than *O*) preventing the formation of side products. We have observed that the sulfonylation reaction proceeds best with two equivalents of hydroxylamine hydrochloride and three equivalents of magnesium oxide in MeOH/H_2_O/THF (3:2:30). The reaction, as monitored by TLC, was complete in only two hours at room temperature. This procedure does not require any aqueous workup and it is potentially scalable, economical and environmentally friendly. *N*-sulfohydroxamic acids are isolated at high yield and purity after a simple filtration [68,69].

### 2.2. EPR Characterization of NO-Donors

**Figure 3:**
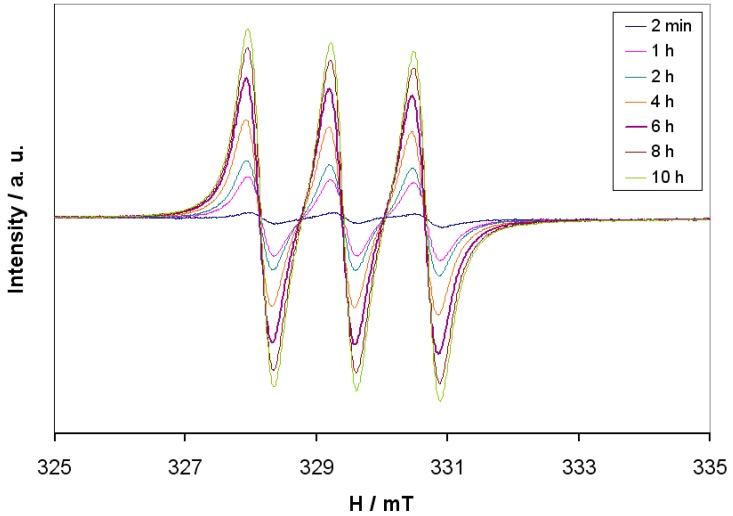
Increase of normalized intensity of EPR signal (measured in arbitrary units) due to Fe(MGD)_2_(NO) as a function of the time in the experiments with PI (0.6 mM in PBS, pH 7.4, 37 °C). All spectra were recorded with the same instrumental parameters.

NO is a diatomic free radical species, hence of paramagnetic nature; it follows that, electron paramagnetic resonance spectroscopy (EPR) is believed the best technique for the direct monitoring of NO in biological matrices [50,52]. The main advantage of EPR in comparison with other NO detection techniques is that it only detects paramagnetic molecules irrespective of the optical appearance of the sample [70] EPR results show that all the compounds studied release NO. Formation of NO is indicated by the three-lines spectrum, due to the coupling between the unpaired electron and ^14^N nucleus (Figure S1 of Supplementary Materials). This indicates the trapping of NO by Fe(MGD)_2_ to give the species Fe(MGD)2(NO) where MGD is *N*-methyl-d-glucamine dithiocarbamate. With elapsing time, the intensity of the signal due to Fe(MGD)_2_(NO) significantly increases, which is correlated with the concentration of NO released by the specific NO-donor (Figure 3). Therefore, EPR spectroscopy can be used to measure the amount and the rate of release of NO by the four compounds studied.

The decomposition of Piloty’s acid and its derivatives is strongly dependent on temperature. In particular, as expected, decomposition under physiological conditions (37 °C) is much faster than at 25 °C (see Figure S2 of Supplementary Materials).

**Figure 4:**
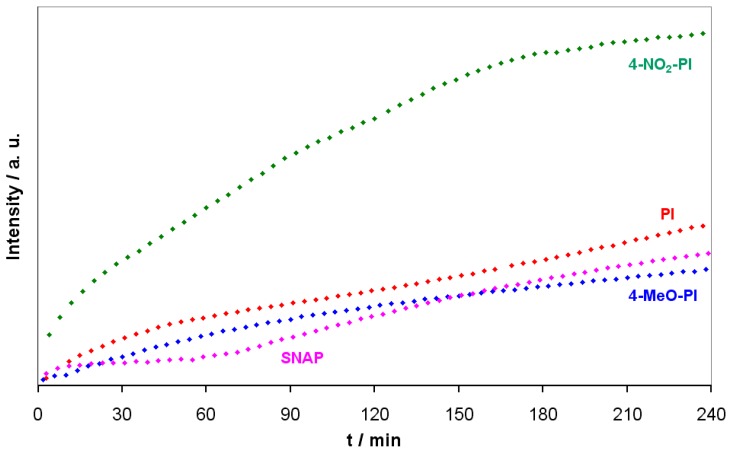
Normalized EPR intensity of the species Fe(MGD)_2_(NO) (measured in arbitrary units) as a function of time for: S-nitroso-*N*-acetylpenicillamine (SNAP), *N*-hydroxy-4-methoxy benzenesulfonamide (4-MeO-PI), Piloty’s acid (PI) and *N*-hydroxy-4-nitrobenzenesulfonamide (4-NO_2_-PI). The concentration of all NO-donors was 0.6 mM. The intensity, obtained after double integration of the spectra, was measured in PBS at 37 °C and pH 7.4. All spectra were recorded with the same instrumental parameters.

In Figure 4 the intensity of NO released by the four NO-donors and trapped by Fe(MGD)_2_ is reported. It is evident that the 4-nitro-derivative (4-NO_2_-PI) releases NO faster and at higher concentrations than Piloty’s acid (PI), while the 4-methoxy substituent (4-MeO-PI) induces a slower release of NO and at lower concentrations. This different chemical behaviour can be attributed to the characteristics of the aromatic ring substituents. The -NO_2_ group is electron withdrawing and increases the rate and the amount of released NO by stabilizing the radical species involved in the decomposition of PI (see Scheme 2) [42]. In contrast, the methoxy substituent is an electron releasing group and disfavours NO release in comparison with the Piloty’s acid parent compound. This trend is confirmed by *N*-hydroxy-4-fluorobenzenesulfonamide which, containing an electron withdrawing substituent, behaves similarly to the nitro derivative [45].

As pointed out in the Introduction, with EPR spectroscopy the HNO eventually formed in the decomposition of PI derivatives cannot be determined. In addition, it is possible to quantify only the total amount of NO formed, but not its origin; in other words, we cannot demonstrate by EPR measurements if all NO revealed comes from the decomposition of PI derivatives or from the oxidation of HNO induced by its coordination to the Fe-based spin trapper.

### 2.3. Electrochemical Characterization of NO-Donors

**Figure 5:**
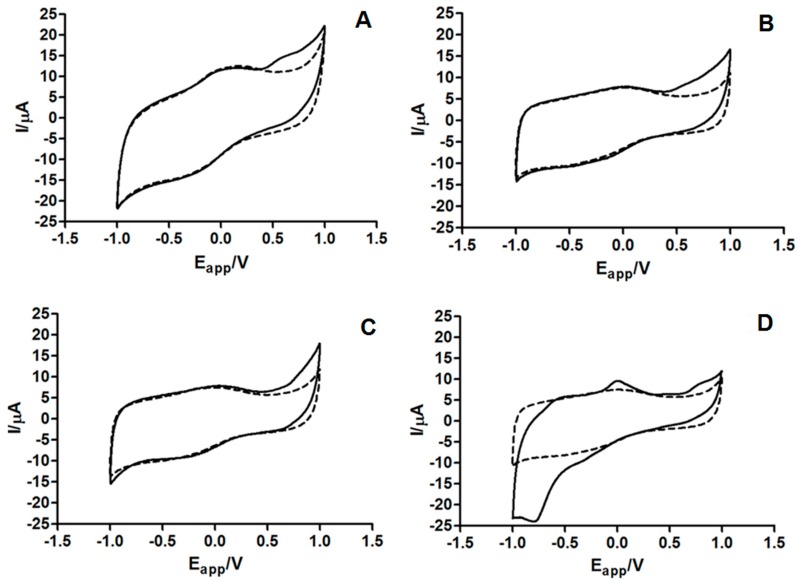
Cyclic voltammograms of SNAP (**A**), PI (**B**), 4-OMe-PI (**C**) and 4-NO_2_-PI (**D**). All experiments were carried out in fresh PBS/DMEM (10%), in the dark, at 37 °C. All voltammograms were recorded in the potential range ±1.0 V vs. Ag/AgCl, using a scan rate of 250 mV s^−1^. Voltammograms shown compare the current from PBS (dashed line) with that of PBS with 1 mM of the respective NO-donor (continuous line).

Figure 5 shows the cyclic voltammograms (CVs) of PBS (dashed line) and NO-donors (continuous lines) at bare epoxy carbon electrodes. All NO-donor molecules resulted in a clear increase of the anodic current around +865 mV. This increase is due to the oxidation of released NO as previously described [71,72]. The absence of a definite peak for NO oxidation could be due to the composition of the transducer material.

### 2.4. In Vitro Calibration of NO Microsensor

**Figure 6:**
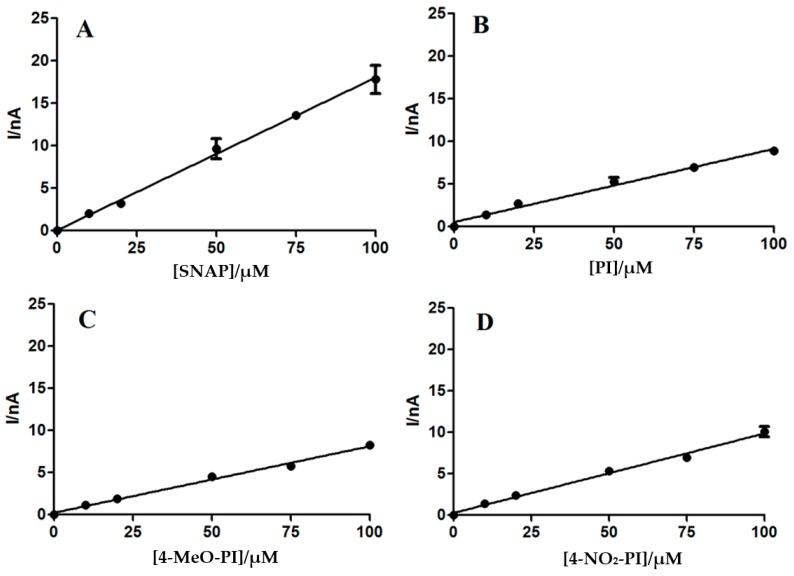
CPAs of SNAP (**A**), PI (**B**), 4-OMe-PI (**C**) and 4-NO_2_-PI (**D**). All experiments were carried out in fresh PBS, in the dark, at 37 °C. Currents were recorded at a fixed potential of +865 mV vs. Ag/AgCl. The concentration range was from 0 to 100 μM for all NO-donors.

As shown in Figure 6, constant potential amperometry (CPA) carried out in PBS/DMEM (10%) with SNAP showed a sensitivity of 0.180 ± 0.005 nA μM^−1^ and R^2^ = 0.997. PI, 4-OMe-PI and 4-NO_2_-PI demonstrated similar results, yielding sensitivities of 0.086 ± 0.004, 0.079 ± 0.003 and 0.096 ± 0.005 nA μM^−1^, respectively, with R^2^ values of 0.987, 0.992 and 0.933, respectively, as shown in Table 1.

The LOD and LOQ values were determined as defined in Section 3.9. Sensors showed a LOD of 38 ± 7 nM and a LOQ of 125 ± 9 nM. Amperometric experiments carried out on Piloty’s acid and its derivatives, under the same conditions as the microdialysis experiments, confirmed that NO is generated as a result of decomposition. Moreover, these molecules are able to liberate NO in the short-term period based on an analysis of the slope values, that reflect the amount of NO released. This finding is in agreement with the EPR experiments. Interestingly, 4-NO_2_-PI was found to release more NO than the other substances tested here.

Surprisingly, the slope of the SNAP CPA was greater than the other substances tested here. This is likely due to the environment where SNAP decomposes. It has been widely demonstrated in the literature that reductive conditions favour the degradation of S-nitrosothiols, such as SNAP [73,74]. In fact, PBS/DMEM (10%) provided a sufficiently reducing environment for the decomposition of SNAP [71].

### 2.5. PC12 Cell Viability

A previous study [71] demonstrated that the effects of NO-donor drugs on DA secretion from PC12 cells in vitro were dependent on the timing of NO generation and the extracellular environment in which NO was generated. The study was performed using in vitro microdialysis of PC12 cell suspensions. This is a suitable in vitro experimental model for studying neurodegenerative diseases which involve a deficiency of dopamine, since PC12 cells are able to synthesize, secrete and metabolize DA. In addition, endogenous NO production in PC12 cells is mediated by neuronal NO synthase (nNOS) which positively modulates DA secretion [27].

**Figure 7:**
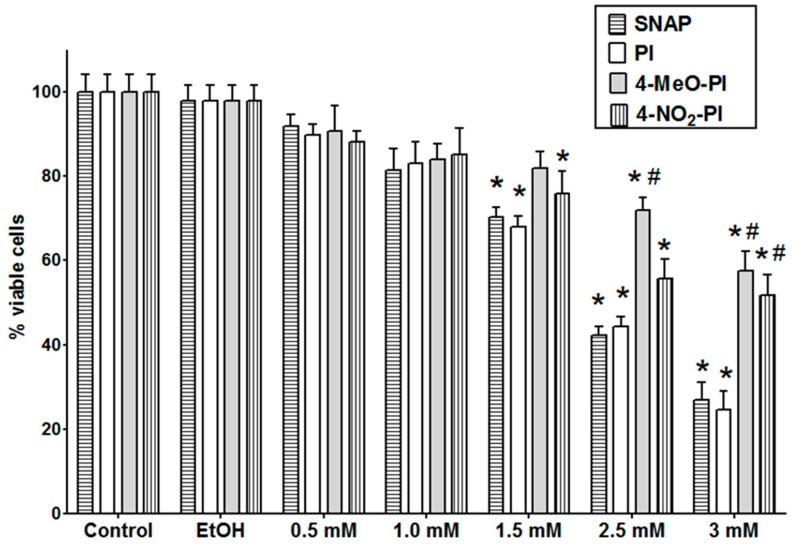
Effects of SNAP, PI, 4-MeO-PI and 4-NO_2_-PI on PC12 cell viability. PC12 cells were incubated in drug-free medium. At the start of each experiment, 100 × 10^3^ cells/cm^2^ were plated and treated 24 h later (time 0) with different concentrations of SNAP, PI, 4-MeO-PI and 4-NO_2_-PI (0.1, 0.5, 1, 1.5, 2 mM). After 24 h, the number of viable cells was determined in triplicate wells. Cell viability was established by the MTT assay. Results are given as percent of control PC12 cells and represent the mean ± SEM of three experiments. * *p* < 0.05 compared with controls; # *p* < 0.05 compared with corresponding ethanol concentrations.

PC12 cells were exposed to varying concentrations of SNAP, PI, 4-MeO-PI and 4-NO_2_-PI at concentrations that did not affect cell viability. As shown in Figure 7, all NO-donors induced a concentration-dependent decrease in cell viability starting from 1.5 mM concentration (*p* < 0.05), as compared to vehicle, with the exception of 4-MeO-PI. From 2.5 mM, further significant differences were observed, where 4-MeO-PI and 4-NO_2_-PI were less toxic than SNAP and PI.

The studies of PC12 viability demonstrated that Piloty’s acid and its derivatives are toxic to cells only at concentrations above 1.0 mM. Furthermore, at higher concentrations, only Piloty’s acid was particularly toxic, showing that nitro- and methoxy-derivatives of PI could be suitable for use in biological models.

In view of these results, Piloty’s acid and its derivatives were used for microdialysis experiments, in order to evaluate their effect on dopamine release from PC12 cell cultures. The cell culture results were comparable with the results from EPR experiments.

### 2.6. Effect of NO-Donor Molecules on Dialysate Levels of DA

**Figure 8:**
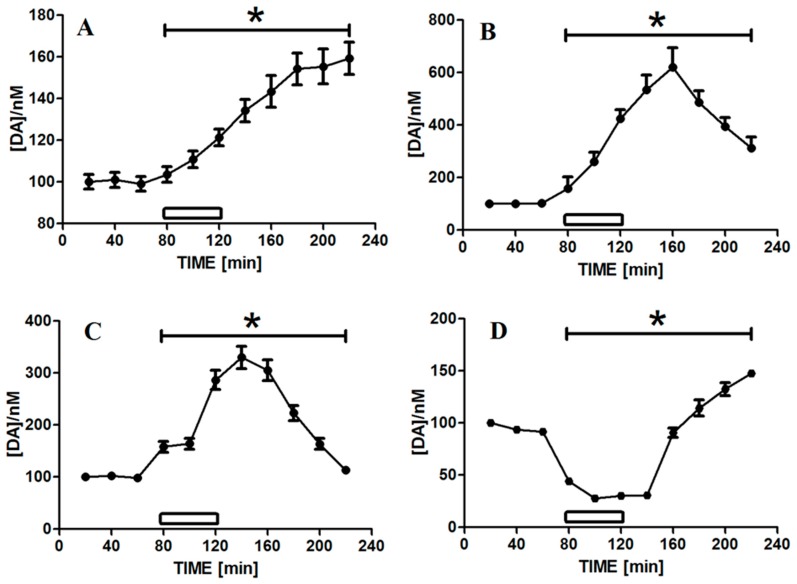
Effect of SNAP (**A**), PI (**B**), 4-MeO-PI (**C**) and 4-NO_2_-PI (**D**) perfusion on DA concentrations in dialysates from PC12 cells suspension. All NO-donors (1.0 mM, *n* = 3 cell suspensions) were infused for 60 min (open bar, 80–120 min). Dialysates were collected, at 20-min intervals before, during and after NO-donor infusion. The collected dialysates were analysed by HPLC. Values are the mean ± S.E.M. * *p* < 0.05 compared with baseline values.

All NO-donors were infused at 1.0 mM for 60 min (*n* = 4 cell suspensions). Data were obtained from the outlet not perfused with the NO-donor. As shown in Figure 8, a 60 min SNAP infusion induced a progressive and significant increase in DA concentration (1.6-fold increase). Under the same conditions, 60 min infusions of PI and 4-MeO-PI induced a statistically significant increase in DA levels (6.2-fold and 3.3-fold, respectively). Conversely, 60 min infusion of 4-NO_2_-PI resulted in a significant decrease in DA concentration (70% decrease as compared to basal levels).

Cell viability was assessed by trypan blue exclusion before filling the capillary apparatus and at the end of each experiment. Non-viable cells were 15.3 ± 1.7%, 13.9 ± 1.6% 3.4 ± 0.7% and 4.4 ± 0.8% in the SNAP, PI, 4-NO_2_-PI and 4-MeO-PI experiments respectively.

As previously demonstrated [71], DA concentrations in the dialysates depended largely on both the timing of NO generation and the extracellular environment in which NO is generated. NO is an unstable, but highly diffusible, simple molecule. In aqueous solution, NO undergoes autoxidation by a third-order rate equation [75]. As previously shown, both the amount and timing of NO generation allowed NO to enter the cells and reach a concentration suitable for activation of the sGC/cyclic GMP pathway [76].

The presence of an electron withdrawing substituent, as in 4-NO_2_-PI, resulted in a rapid and consistent release of NO. Infusion of 4-NO_2_-PI resulted in a significant decrease in DA, of more than 70% compared to basal levels. Most likely, this decrease reflects that a greater amount of NO diffuses across the cell membrane than either undergoes extracellular autoxidation [74] and/or induces extracellular DA nitration [77,78], with a consequent decrease in dialysate DA concentration.

Conversely, in agreement with EPR results, the presence of an electron-donor group, as in 4-MeO-PI, resulted in a slow release of NO. Infusion of 4-MeO-PI resulted in a significant and long-lasting increase in dialysate DA levels, confirming that the timing, and in some ways the amount, of NO generation influences DA release from cells.

Piloty’s acid had a similar effect to 4-MeO-PI, but resulted in a greater increase in dialysate DA levels. This greater increase could reflect the balance between DA release, due to NO passage through the cell membrane, and that arising from cell death due to the toxicity of PI.

From the microdialysis experiments, it is possible infer the toxicity of the molecules tested as the DA dialysate content results from both release due to NO stimulation, as well as lysis due to cell death. DA release measured with perfusion of SNAP and 4-NO_2_-PI increased and did not return to basal levels. This could be due to the toxicity of these compounds, as demonstrated by the trypan blue exclusion results. MTT data at 24 h showed that SNAP is toxic, while 4-NO_2_-PI seems to be less toxic over the same time frame.

In relation to DA release, Piloty’s acid and 4-MeO-PI were less toxic in the short term as DA in the dialysates approached baseline values. The higher amount of DA release with Piloty’s acid perfusion could be due to the relatively higher toxicity observed, even in long-time viability experiments.

These results can be applied to the development of new NO-donor drugs for neurodegenerative pathologies, involving a deficiency of dopamine and where an inflammatory component is overt, such as Parkinson’s disease. Therapies for such diseases should be able to slowly release NO at concentrations that allow entry of NO into the cells resulting in DA release, but that reduce DA auto-oxidation or cell death.

## 3. Materials and Methods

### 3.1. Chemicals

Starting materials, reagents, and dry solvent were purchased from Sigma-Aldrich (Milano, Italy) and used without further purification. Insulated copper wires were purchased from Advent Research Materials, Suffolk, U.K. Thin layer chromatography (TLC) was performed on Merck Kieselgel 60 TLC plates. ^1^H NMR and ^13^C NMR spectra were recorded in the solvents indicated at 300 MHz and 75 MHz, respectively, unless otherwise noted. Chemical shifts are reported in parts per million (ppm, δ), were measured from Tetramethylsilane (0.0 ppm) and are referenced to the solvent CDCl_3_ (7.26 ppm), DMSO-d_6_ (2.49 ppm), for ^1^H NMR, and CDCl_3_ (77.0 ppm), DMSO-d_6_ (39.5 ppm) for ^13^C NMR. Data are reported as follows: chemical shifts, multiplicity (s = singlet, d= doublet, t = triplet, q = quartet, br = broad, brs = broad singlet, m = multiplet, *p* = pseudo), coupling constants J (Hz), relative integration value. High resolution mass spectra (HRMS) were obtained using electron-impact (EI) or electrospray ionization (ESI). All newly synthesized compounds gave satisfactory elemental analysis. HPLC analysis was performed on a Waters Alliance HT 2795 Separation Module with a Waters 2996 Photodiode Array Detector using an Xterra MS C-18 column (5 μM, 4.6 × 150 mm) eluting with aqueous acetonitrile (*v/v* 1/1) containing 0.05% CF3COOH at a flow rate of 1 mL/min. Melting points were recorded in open capillary tubes using a Buchi apparatus and the values reported are uncorrected.

### 3.2. Organic Synthesis

#### 3.2.1. Synthesis of *N*-Methyl-d-glucamine Dithiocarbamate (MGD)

MGD was synthesized according to an established procedure [79,80]. A solution of sodium hydroxide 2.5 N was prepared by dissolving NaOH pellets (20 g, 0.5 mol) in distilled water (200 mL) at 0 °C. The reaction vessel containing sodium hydroxide solution was cooled with an ice/salt bath, and then *N*-methyl-d-glucamine (NMG) (97.6 g, 0.5 mol) was added dropwise to the alkaline solution and dissolved with stirring. The solution was warmed from −5 °C to 5 °C. A separate solution of carbon disulfide (CS_2,_ 150 mL, 0.83 mol) in absolute ethanol (50 mL) was prepared and cooled to 0 °C. The cold solution of CS_2_ was slowly added into the previously prepared cold solution of NMG in NaOH with stirring over a period of about 15 min. The temperature of the reaction mixture was kept between 0 °C and 5 °C during the carbon disulfide addition to minimize the formation of coloured side products. After addition of the CS_2_ solution, cooling and stirring of the reaction mixture was continued for an additional 20 min. Also, after addition of the CS_2_ solution, MeOH (500 mL) was added and the vessel covered to prevent solvent evaporation. The mixture was allowed to stand overnight to facilitate the formation of larger crystals. The MGD precipitate was collected by vacuum filtration. The filter cake was washed with solutions of ethanol with progressively higher ethanol contents, as follows: first, with 70% ethanol (500 mL), then with of 95% ethanol (1000 mL), and then with absolute ethanol (500 mL). To dry the collected precipitate, the vacuum was maintained for 2 h, followed by air drying to assure desiccation. The filtrate and washings were refrigerated to obtain a second crop of crystals. The first crop of precipitate air dried to a white powder with a yield of 107.4 g (69%). The second crop of precipitate was slightly yellow in colour with a yield of 17.1 g (11%). Analysis of this powder indicated that it was a monohydrate form. The melting point was 231–232 °C, which is consistent with the melting point of commercially available MGD (232–233 °C). The experimental elemental analysis results (C, 30.74; H, 5.90; N, 4.54) were consistent with the expected values (C, 30.86; H, 5.82; N, 4.49) for C_8_H_18_NO_6_S_2_Na^+^

#### 3.2.2. Synthesis of *N*-Hydroxy-4-methoxybenzenesulfonamide

Hydroxylamine hydrochloride (0.72 g, 10 mmol) in MeOH/H_2_O (3:2, 5 mL) was treated with MgO (0.34 g, 8.6 mmol), followed by a solution of *p*-Methoxybenzenesulfonyl chloride (0.89 g, 4.3 mmol) in THF (30 mL), and then MgO (0.17 g, 4.3 mmol). The reaction was vigorously stirred at room temperature until the sulfonyl chloride had completely disappeared, as determined by TLC (EtOAc–hexane, 1:1; 2 h). Then, the mixture was filtered through a pad of Celite first, and then on a short plug of silica gel. The clear filtrate was dried over MgSO_4_ and evaporated to dryness to give the resulting *N*-hydroxy-4-methoxybenzenesulfonamide (0.81 g, 93%) as a crystalline white solid (99% purity). The melting point of the isolated solid was 123–127 °C, which is consistent with the literature value (126–128 °C) [81]. ^1^H NMR results are shown in Figure S3 of the Supplementary Materials (DMSO-d_6_, δ = 9.52 (d, *J* = 3.0 Hz, 1H), 9.40 (d, *J* = 3.0 Hz, 1H), 7.78 (d, *J* = 9.0 Hz, 2H), 7.13 (d, *J* = 9.0 Hz, 2H), 3.82 (s, 3H)). ^13^C NMR results are shown in Figure S4 of the Supplementary Materials (CDCl_3_, δ =163.5, 131.0, 129.6, 114.8, 56.4). IR (thin film) analysis gave the following results: ν 3440, 2107, 1640, 1491, 1452, 1321, 1250, 1094, 1015 cm^−1^. HRMS (ES^+^) experimental results (MNa^+^: 226.0144) were consistent with the calculated expected results for C_7_H_9_NO_4_SNa^+^ (226.0144). The experimental elemental analysis results (C, 41.40; H, 4.52; N, 6.95) were consistent with the expected results for C_7_H_9_NO_4_S (C, 41.37; H, 4.46; N, 6.89).

#### 3.2.3. Synthesis of *N*-Hydroxy-4-nitrobenzenesulfonamide

*N*-hydroxy-4-nitrobenzenesulfonamide was prepared as previously reported [68]. A crystalline yellow solid was obtained at 97% yield (98% purity), with a melting point of 158–160 °C, which is consistent with that reported in the literature (154–155 °C) [82]. ^1^H NMR results are shown in Figure S5 of the Supplementary Materials (CDCl_3_, δ = 9.69 (s, 1H), 9.52 (s, 1H), 8.37 (d, *J* = 8.95, 2H), 8.16 (d, *J* = 8.93 Hz, 2H)). ^13^C NMR results are shown in Figure S6 of the Supplementary Materials (CDCl_3_, δ = 149.4, 142.56 129.2, 122.9). The experimental elemental analysis results (C, 33.10; H, 2.86; N, 12.75) were consistent with the expected results for C_6_H_6_N_2_O_5_S (C, 33.03; H, 2.77; N, 12.84). HRMS (ES^+^) experimental results (MNa^+^: 240.9887) were consistent with the calculated expected results for C_6_H_6_N_2_O_5_SNa^+^ (240.9890).

### 3.3. Electron Paramagnetic Spectroscopy

#### 3.3.1. Theory Background and Spin Trapping

**Figure 9:**
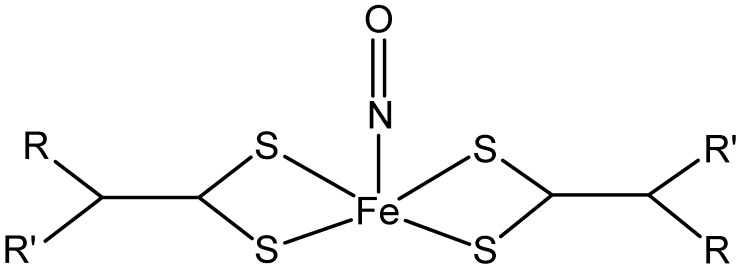
Structure of the complex Fe(MGD)_2_(NO).

EPR (Electron Paramagnetic Resonance) is a resonance spectroscopy, which can be applied to paramagnetic species (i.e., chemical species with unpaired electrons), such as NO [83]. In the absence of any hyperfine coupling with other nuclei, only a transition (recorded as the first derivative of the absorption curve) is expected. Instead, when an unpaired electron couples with a nucleus with angular momentum of nuclear spin *I*, (2*I* + 1) signals with identical intensity are expected. Although NO is a paramagnetic species, it cannot be detected directly using EPR spectroscopy because of its high reactivity and very short lifetime in aqueous solution. Therefore, spin trapping must be used. The spin trap is a chemical species that reacts with the short-lived NO radical to form a relatively stable free radical. One of the first spin traps for NO was deoxyhemoglobin [84]. Subsequently, Mordvintcev et al., proposed the use of Fe^2+^ complexes with diethyldithiocarbamate [85], but this approach is limited by their poor solubility in water. Komarov et al., improved the technique by using a species of Fe^2+^ with *N*-methyl-d-glucamine dithiocarbamate (Fe(MGD)_2_), which is soluble in water [86]. In this work, we used Fe(MGD)_2_ as the spin trapping agent. NO released by the decomposition of the NO-donors is trapped by Fe(MGD)_2_, forming iron-nitroxyl species (Fe(MGD)_2_(NO)) (Figure 9). This reaction results in a stable form of NO that is easily detectable as a three-line EPR signal, arising from the hyperfine coupling between the unpaired electron and ^14^N nucleus (*I* = 1). The intensity ratio between the three resonances is 1:1:1 and the value of the hyperfine splitting constant, *a*(^14^N) is 12.5 G (Figure S1 of Supplementary Materials).

The intensity of the EPR signal due to the trapped NO increases as a function of time as the concentration of the species Fe(MGD)_2_(NO) increases (see Figure 4). Therefore, EPR spectroscopy can be used to measure the amount and the rate of NO release from a series of NO-donors. It must be observed that under the experimental conditions used in this study PI derivatives can release also HNO as discussed in the Introduction [44,45].

#### 3.3.2. Experimental

EPR spectra were recorded in aqueous solutions with an X-band (9.40 GHz) Bruker EMX spectrometer. A solution of Fe(MGD)_2_ was prepared in PBS, by dissolving MGD (0.045 M) and FeSO_4_ ion (FeSO_4_, 0.015 M). Solutions containing Piloty’s acid were prepared in DMSO or H_2_O using a ratio 1:1 with Fe(MGD)_2_. Different concentrations of PI and Fe(MGD)_2_ were used (0.12, 0.20, 0.50, 0.60, 0.90 and 1.20 mM). Approximately 1 mL of solution was kept with a Pasteur pipette, and subsequently inserted in the cavity for EPR analysis. The acquisition time between spectra was 4 min and the total duration of the experiments was between 1 and 10 h. Experiments were carried out at room temperature (25 °C) or physiological temperature (37 °C). An identical procedure was followed for the PI derivatives, 4-NO_2_-PI and 4-OMe-PI. The results were compared with those for SNAP in H_2_O. The instrumental EPR parameters were as follows: modulation frequency 100 kHz, modulation amplitude 3 Gauss, sweep time 168 s, microwave power 20 mW; this microwave power, using the Bruker ER 4119HS resonator, is below the saturation level. The spectrum in Figure S1 was simulated with the computer program WinEPR SimFonia 1.25 (Bruker Analytische Messtechnik GmbH) with the following parameters *a*(^14^N) 12.5 G, *g* 2.0428, linewidth 4 G, Lorentzian/Gaussian 0.10. The intensity of EPR signals (Figure 2 and Figure S2) was estimated after double integration of the spectra.

### 3.4. NO Microsensors

#### 3.4.1. Preparation of NO Microsensors

**Figure 10:**
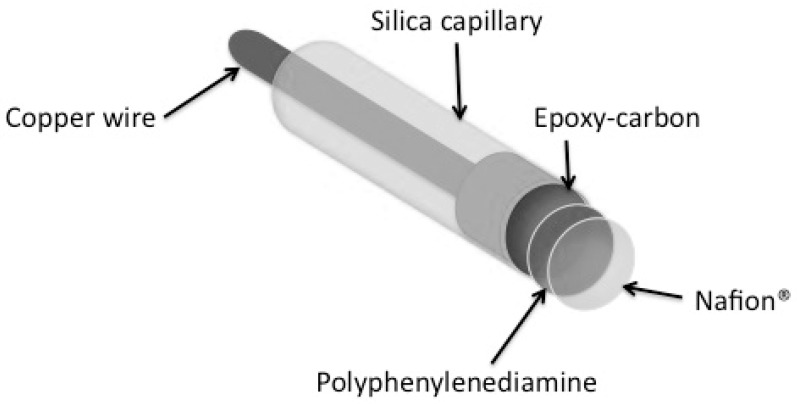
Scheme of NO microsensor. The active surface is a 180 μm diameter epoxy-carbon disk. The disk surface is then modified with p-OPD and Nafion^®^ to ensure selectivity of the sensors for NO.

NO microsensors (Figure 10) were made using insulated silver wires (50 mm in length) using a modified approach of previously described procedures [87,88]. Approximately 3.0 mm of silver wire was exposed at both ends of the wire and one end was inserted into a silica capillary tube (20 mm in length; i.d. 180 μm, Polymicro Technologies, Phoenix, AZ, USA). A 180 μm diameter carbon-composite disk electrode (area: 2.5 × 10^−4^ cm^2^) was then fabricated by mixing 840 mg of graphite with 500 mg of Araldite-M and 200 mg of hardener and filling the silica capillary tubing with the mixture. After 24 h at 40 °C, the sensor surface was polished using a high speed drill (Dremel^®^ 300, Dremel Europe, Breda, the Nederlands) equipped with an aluminum oxide grinding wheel [60]. The surface of the fabricated sensors was then polished with alumina. These bare electrodes were used for a preliminary electrochemical characterization of NO-donors by cyclic voltammetry.

The sensors were then modified with O-phenylenediamine (OPD) and Nafion^®^ using a combination of previously described protocols [26,89,90,91,92,93] in order to make them selective for NO and to avoid electrochemical responses from the most common physiological, electroactive molecules. After fabrication, the electrodes were characterized and only electrodes with NO detection limits <50 nM and selectivity against ascorbic acid (>1000:1), dopamine (>250:1), and nitrite (>800:1) were used [59].

#### 3.4.2. Microsensor Characterization of NO Donors

Cyclic voltammetry and constant potential amperometry, were used in addition to EPR experiments, in order to confirm the generation of NO by the compounds under study. As previously described [88], a three-electrode electrochemical cell was used for both electropolymerisation of OPD and electrochemical measurements of NO release. All amperometric experiments were carried out by connecting epoxy-carbon microsensors, as working electrodes (WE), Ag/AgCl (NaCl, 3 M) as a reference electrode (RE) and a high surface area stainless steel electrode as an auxiliary electrode (AE), to a four-channel equipment (eDAQ QuadStat, e-Corder 410, eDAQ, 6 Doig Ave, Denistone East NSW 2112, Australia).

Cyclic voltammetry (CV) was carried out in order to investigate the redox properties of the newly synthetized NO-donors in a buffered aqueous solution (PBS) at physiological conditions (pH 7.4, 37 °C) and for confirming the oxidation of released nitric oxide. Voltammograms were recorded in the potential range ±1.0 V using a scan rate of 250 mV s^−1^. Bare epoxy carbon electrodes were used during acquisition of CVs to obtain a complete electrochemical profile of the NO-donors. SNAP was used as reference molecule for comparison with the other NO-donor molecules studied here. SNAP CVs were obtained in the dark at 37 °C in fresh PBS over a concentration range between 0.5 and 2.5 mM (Figure 3). NO-donor solutions were freshly prepared immediately before the experiments by dissolving the compounds in DMSO to obtain 100 mM solutions. These stock solutions were injected into the experimental cells to obtain final concentrations between 0.5 and 2.5 mM. For amperometric calibrations, OPD/Nafion modified electrodes were used.

SNAP was also used as a reference compound for constant potential amperometry (CPA) as the decomposition kinetics and yield of NO are well characterized. SNAP was used as a standard for microsensor calibration by measuring the relative NO release in a CuCl saturated solution in the dark by applying a constant potential of +865 mV vs. Ag/AgCl and adding known aliquots of SNAP stock solution for final concentrations from 0 to 2.5 μM [91].

CPAs for all NO-donor molecules were carried out in fresh PBS/DMEM 10% (see Section 3.7) and under physiological conditions (pH = 7.4 and 37 °C) in order to assess the yield of released NO compared to SNAP. A constant potential of +865 mV vs. Ag/AgCl was applied and, after a stable baseline was reached, known amounts of NO-donor stock solutions were added to the PBS in order to obtain final concentrations between 0 and 100 μM.

### 3.5. Cell Culture and Capillary Tube Construction for In Vitro Microdialysis

PC12 cells were maintained at 37 °C in 60-mm plastic culture plates in Dulbecco’s modified Eagle’s medium (DMEM)/F12 supplemented with 10% horse serum and 5% fetal calf serum, in a humidified atmosphere of 5% CO_2_/95% air [94]. After short-term culture (15–20 passages), the cells were washed twice in 2 mL of phosphate buffered saline PBS/DMEM 10% containing glucose (10 mM), NaCl (137 mM), CaCl_2_ (1.17 mM) and MgCl_2_ (1.0 mM), harvested by trituration and centrifuged (94 g for 5 min). Cells were re-suspended in PBS/DMEM and the number of cells/50 μL was assessed using trypan blue in a Burker’s chamber. The initial volume of the cell suspension was adjusted to reach a final concentration of 900 × 10^3^ cells/50 μL.

The capillary tube for cell culture suspension microdialysis has been described in detail in a previous study [27,94,95]. Briefly, the probe was made using two sections of plastic-coated silica tubing (o.d. 150 μm, i.d. 75 μm, Scientific Glass Engineering, Milton Keynes, UK), each placed in the center of a semi-permeable, polyacrylonitrile dialysis fiber (AN-69; Hospal Industrie, Meyzieu CEDEX, France, 45 mm length). Each semi-permeable membrane had an active length of 40 mm. Thereafter, each section of plastic-coated silica tubing was positioned in the center of a section of polythene tubing (i.d. 0.58 mm; Portex, Hythe, Kent, UK, 35 mm length). The two sections of silica tubing served as inlets for connections with the dialysis pump. Dialysates from each polyacrylonitrile dialysis fiber were collected from the sections of polythene tubing, which served as outlets. A third section of plastic-coated silica tubing (40 mm length) was sealed outside the polythene tubings to permit the aspiration of PC12 cell suspension into the microdialysis probe. All probe components were coated with quick drying epoxy glue. Thereafter, the microdialysis apparatus (semi-permeable polyacrylonitrile dialysis fibers positioned side-by-side plus sealed plastic coated silica tubing) was placed in an un-heparinized micro-hematocrit capillary tube (7.5 mm length, 1.1 mm i.d.; Chase Scientific Glass Inc., Rockwood, TN, USA), having a volume of 70 μL. The side-by-side positioning of the dialysis fibers permits separate co-infusion of drugs. In addition, the two distinct outlets permit separate dialysate sample collection from the same PC12 cell suspension. Separate sample collection is useful when, for example, one or more drugs which may have either pro-oxidant or antioxidant properties are infused at the same time.

### 3.6. Microdialysis Procedure

The microdialysis procedure has been described in detail previously [94,95]. Briefly, the cellular microdialysis probe was perfused with PBS/DMEM 10% by means of a peristaltic microinfusion double channel pump (P720 peristaltic pump; Instech, Plymouth Meeting, PA, USA) which pumped PBS/DMEM from a 60 mm plastic culture plate in an IR incubator at 37 °C in a humidified atmosphere of 5% CO_2_/95% air at a flow rate of 3.0 μL/min. The pump channels were connected to the probe inlets by a length of polythene tubing. The perfusion apparatus was then filled with 50 μL of the PC12 cell suspension by manual aspiration, using a 1.0 mL syringe connected to the plastic coated silica tubing sealed outside the polythene tubings. Thereafter, the perfusion apparatus was kept at 37 °C. Samples were collected manually in 250 μL micro-centrifuge tubes (Alpha Laboratories, Eastleigh, UK) from both outlets. Then, a 30 μL aliquot of the dialysate, recovered from the probe outlet not perfused with the NO-donor was analysed by HPLC. The first sample was collected after 60 min of stabilization (time 0). After this, dialysates were collected at 20-min intervals, for 40 min prior to the infusion of NO-donors. The concentration of dopamine detected after the first 20 min of perfusion was taken as the concentration at time 0. No significant decrease in cell viability was observed at the end of the experiments, as previously observed [27,94,95]. Cell viability was assessed before filling the capillary apparatus and at the end of each experiment by trypan blue exclusion. For each experiment, the cell viability was given as the difference between final and initial percentages of non-viable cells.

### 3.7. Chromatographic Analysis of Dialysates from PC12 Cell Suspension

DA was quantified by high performance liquid chromatography coupled with electrochemical detection (HPLC-EC), as previously described [27,94], using an Alltech 426 HPLC pump equipped with a Rheodyne injector (model 7725), a C18 column (150 mm × 4.6 mm i.d., Thoso Haas ODS80TM C18, Im Leuschnerpark 4D-64347 Griesheim, Germany ), an electrochemical detector (BAS modeldeleted LC4B, 2701 Kent Avenue West Lafayette, IN, USA) and a PC-based analog-to-digital converter system (Varian Star Chromatographic Workstation, Valnut Creek, CA, USA). The mobile phase was citric acid (0.1 M), sodium acetate (0.1 M), EDTA (1.0 mM), MeOH (9%) and sodium octylsulphate (50 mg/L) at pH = 2.9. The flow rate was 1.3 mL/min.

### 3.8. Assessment of Cell Viability

Cell viability was determined with the 3-(4,5-dimethylthiazol-2-yl)-2,5-diphenyltetrazolium bromide (MTT) assay after 24 h of incubation. In the MTT assay, viable cells convert the soluble dye MTT to insoluble blue formazan crystals. At the start of each experiment and at selected intervals thereafter, the cell number was determined in triplicate wells. In brief, 1 mg of MTT was added per mL of medium, and the cultures were incubated at 37 °C for 4 h. The cells were rinsed with PBS and centrifuged at 4000 rpm for 20 min to remove the supernatant containing MTT. Thereafter, the supernatant was discarded and the pellet was dissolved in 2 mL of isopropanol and centrifuged at 4000 rpm for 5 min. The absorbance of the supernatant was then determined at 600 nm using a Bauty Diagnostic microplate reader. A standard curve was constructed utilizing known concentrations of cells at the start of every experiment.

### 3.9. Statistical Analysis

Concentrations of DA in dialysates from PC12 cell suspension are reported in nM and given as the mean ± SEM. Electrochemical currents are reported in nA while concentrations of NO generated from NO-donors in PBS are reported in μM. NO-donor effects on DA were statistically evaluated in terms of changes in absolute dialysate concentrations. Statistical significance was assessed using an ANOVA with Newman-Keuls post-hoc analysis. The null hypothesis was rejected when *p* < 0.05.

Limits of detection and quantification (LOD and LOQ, Equations (1) and (2)) have been calculated by means of a statistical method based on the standard deviation (σ) of the response and the linear region slope (LRS) of the calibration curve [96] as follows:

LOD = 3.3σ/LRS
(1)

LOQ = 10σ/LRS
(2)

## 4. Conclusions

In this study the NO release from Piloty’s acid and its derivatives (4-NO_2_-PI and 4-OMe-PI) with different electronic properties have been investigated, and the results have been compared with a benchmark NO-donor (SNAP). An efficient multigram synthesis procedure was developed that allows preparation of a sulfohydroxamic acid library without any tedious purification steps associated with previously published strategies. The synthetic methods presented here can be, in principle, applied to the preparation of any Piloty’s acid derivative. NO release was investigated using spectroscopic and electrochemical techniques. EPR spectroscopy used in spin trapping experiments with Fe(MGD)_2_ certainly detects NO, but it is difficult to establish with certainly if the HNO eventually released by the PI derivatives can be coordinated to Fe(II) favouring its oxidation to NO and therefore resulting in the contemporaneous detection of both these species. The results of EPR spectroscopy should be combined with those obtained with other techniques, such as the electrochemical studies [97]. Experimental data showed that Piloty’s acid derivatives with electron-withdrawing substituents favoured NO generation in physiological conditions, while electron-donor groups disfavoured this process. However, in the light of the results of the tests on the dopamine release from PC12 cells induced by PI derivatives and of the potential application in the therapy of Parkinson’s disease, we would like to highlight here that we are interested not in the detailed mechanism of decomposition of these compounds, but in the effect of the total NO generated (i.e., formed directly by the decomposition of PI derivatives or indirectly by the oxidation of HNO) in biological systems.

In this regard, 4-MeO-PI and, probably, all derivatives with electron-donor groups on the aromatic ring could represent an interesting class of NO donors, because PI derivatives are not toxic over the concentration range used here and decomposes to yield NO at a rate that allows the passage of NO through the membranes and the release of dopamine, but that does not induce considerable cell damage or death. Based on these preliminary data, we intend to design and synthesize new Piloty’s acid derivatives with electron-donating groups on the phenyl ring and evaluate the dopamine release induced by these molecules. Although the potential anti-inflammatory properties of the newly-synthesized molecules were not investigated in this study, these will be characterized in future research as Cyclo-oxygenase Inhibitor Nitric Oxide Donors (CINODs) have recently been shown to be effective in the treatment of neurodegenerative diseases [98,99], such as Alzheimer’s and Parkinson’s disease.

## Figures and Tables

**Table 1:** In Vitro NO calibration parameters (slope ± SEM and R^2^) obtained from NO-donor molecules on OPD/Nafion^®^ (The Chemours Company FC, LLC) modified epoxy carbon electrodes *^a^*.

NO-Donor	Slope (nA μM^−1^) ± SEM	R^2^	NO Released (μM mM^−1^_donor_) *^b^*
SNAP	0.180 ± 0.005	0.997	14.0
PI	0.086 ± 0.004	0.987	7.7
4-MeO-PI	0.079 ± 0.003	0.992	6.9
4-NO_2_-PI	0.096 ± 0.005	0.993	8.4

*^a^* Data were obtained by applying a constant potential of +865 mV, in the dark at 37 °C. *^b^* The NO released was calculated on the basis of decomposition of SNAP in CuCl, given a 90% NO yield (see ref. [41]) for 1 mM of NO-donor.

## Supplementary Materials

The following are available online at www.mdpi.com/1424-8247/10/3/74/s1, Figure S1: Experimental (in blue) and simulated (in pink) spectra of the species, Figure S2: EPR intensity of the species Fe(MGD)_2_(NO), Figure S3: ^1^H NMR of *N*-hydroxy-4-methoxybenzenesulfonamide, Figure S4: ^13^C NMR of *N*-hydroxy-4-methoxybenzenesulfonamide, Figure S5: ^1^H NMR of *N*-hydroxy-4-nitrobenzenesulfonamide, Figure S6: ^13^C NMR of *N*-hydroxy-4-nitrobenzenesulfonamide.
