# Supplementary material for: Synthesis of Nitric Oxide Donors Derived from Piloty’s Acid and Study of Their Effects on Dopamine Secretion from PC12 Cells
Source: Pharmaceuticals (Basel). 2017 Sep 5;10(3):74. doi: 10.3390/ph10030074 (PMC5620618; doi:10.3390/ph10030074)
Supplement: Supplementary file 1 [file pharmaceuticals-10-00074-s001.pdf]

Synthesis of Nitric Oxide Donors Derived from Piloty's Acid and Study of their Effects on Dopamine Secretion from PC12 cells

Daniele Sanna [1], Gaia Rocchitta [2], Maria Serra [1], Marcello Abbondio [3], Pier Andrea Serra [2,*], Rossana Migheli [2], Lidia De Luca [4], Eugenio Garribba [4,*] and Andrea Porcheddu [5,*]

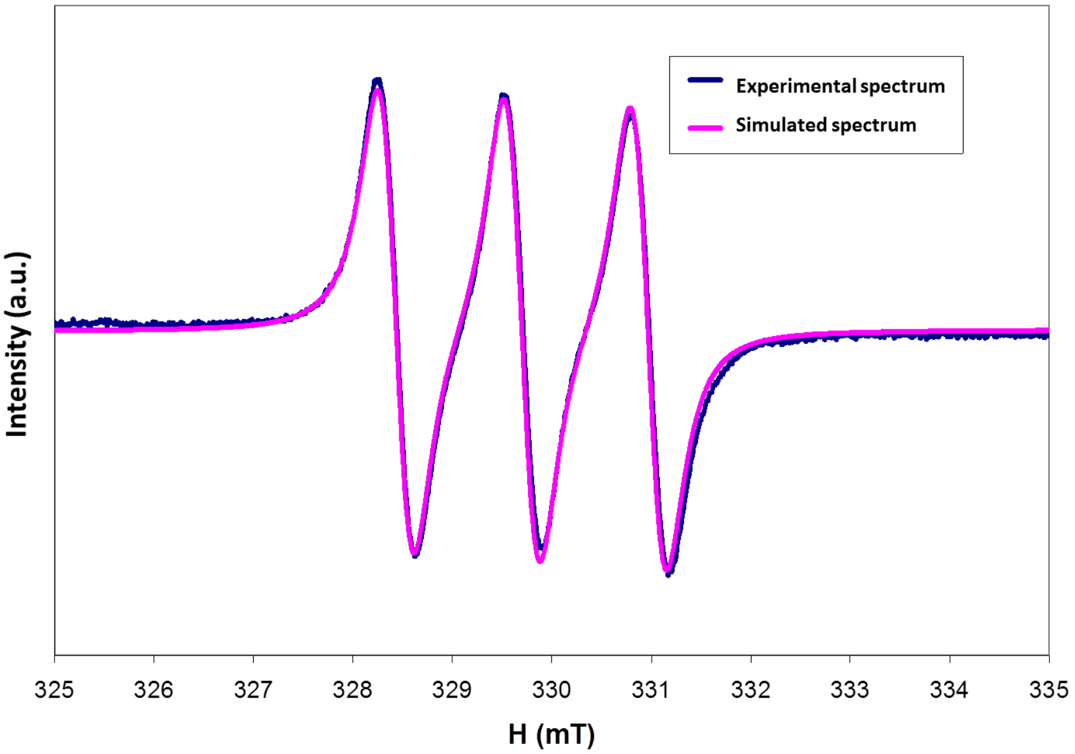

Figure S1. Experimental (in blue) and simulated (in pink) spectra of the species $Fe(MGD)_2(NO)$, obtained after the interaction of NO with the spin trapped $Fe(MGD)_2$.

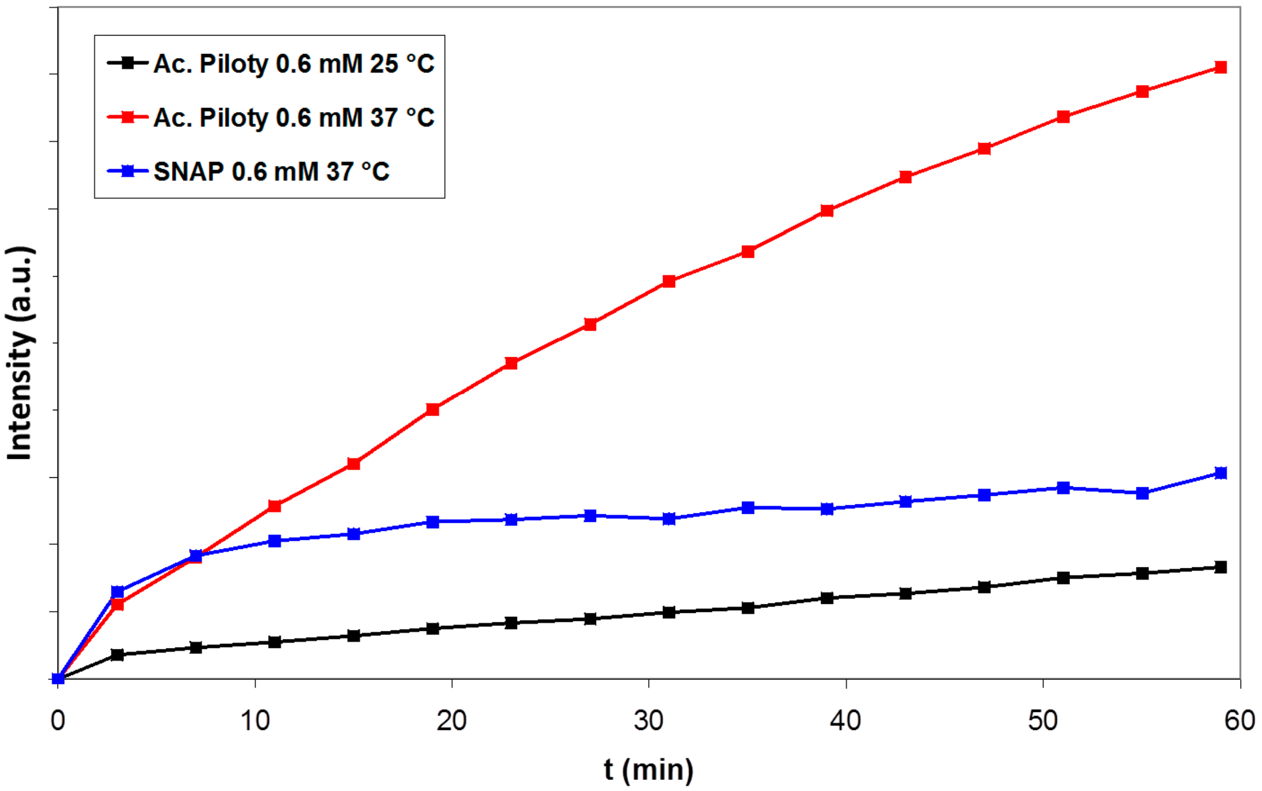

Figure S2. EPR intensity of the species $Fe(MGD)_2(NO)$ (measured in arbitrary units) as a function of the time for Piloty's acid (at 25 and 37 °C) and SNAP (at 37 °C).

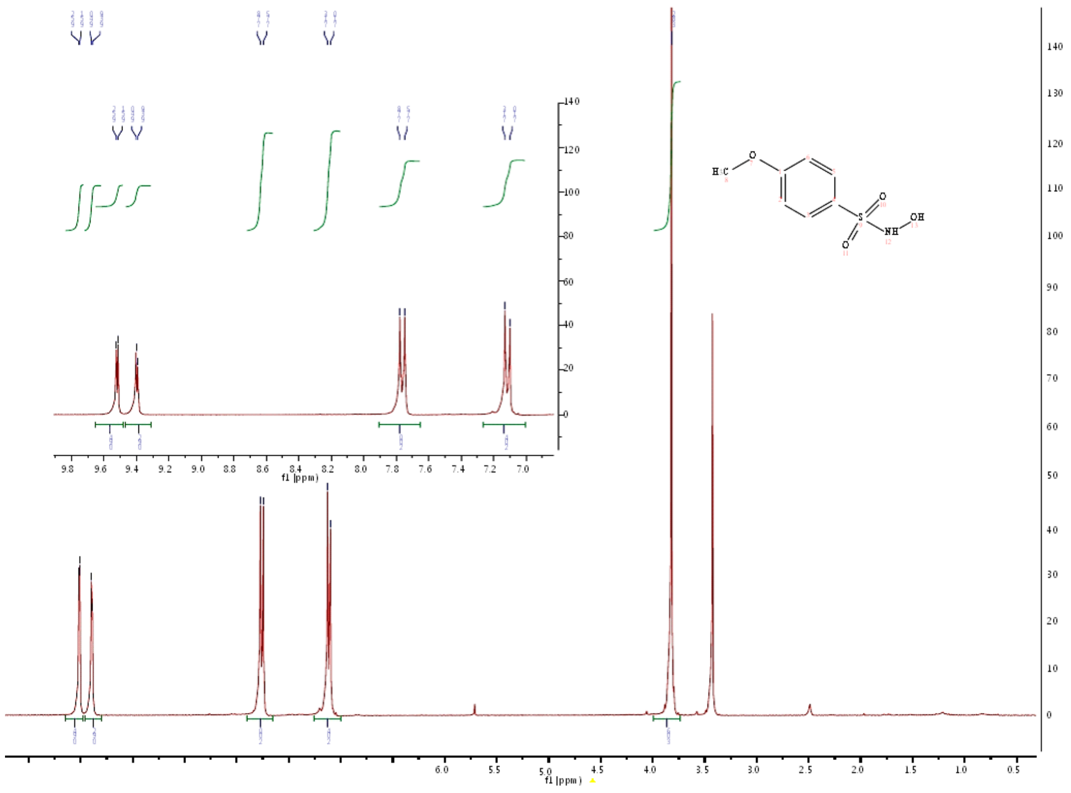

Figure S3. ^{1}H NMR of N-hydroxy-4-methoxybenzenesulfonamide.

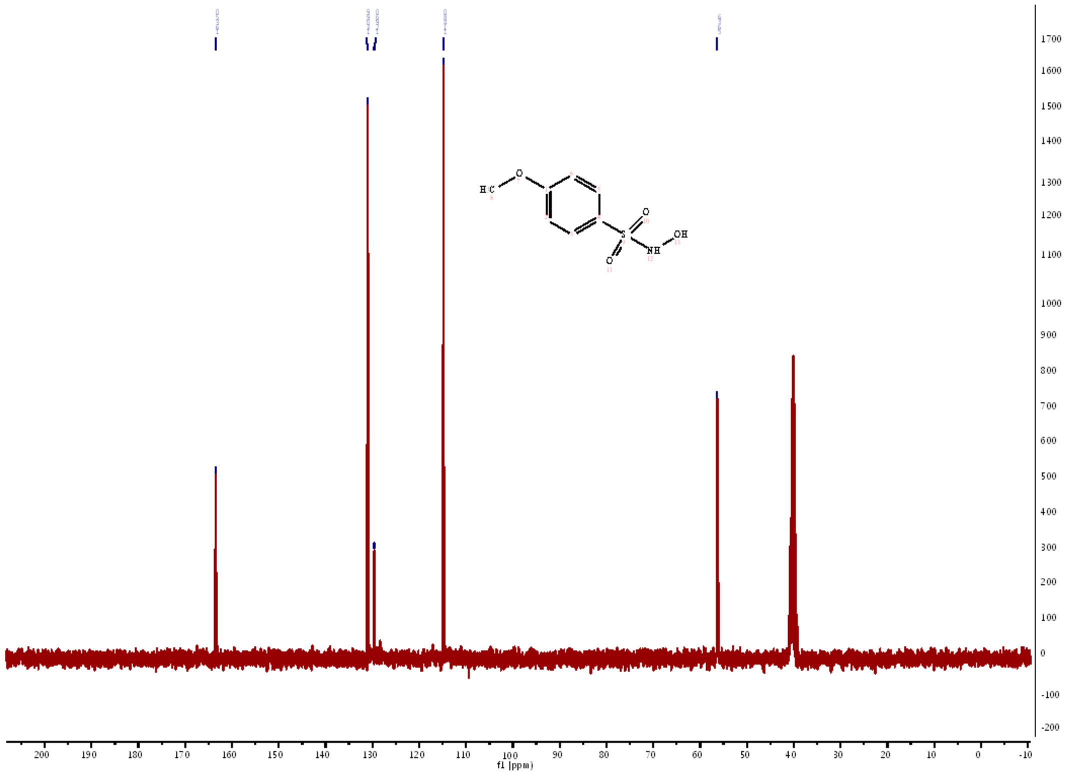

Figure S4. ^{13}C NMR of N-hydroxy-4-methoxybenzenesulfonamide.

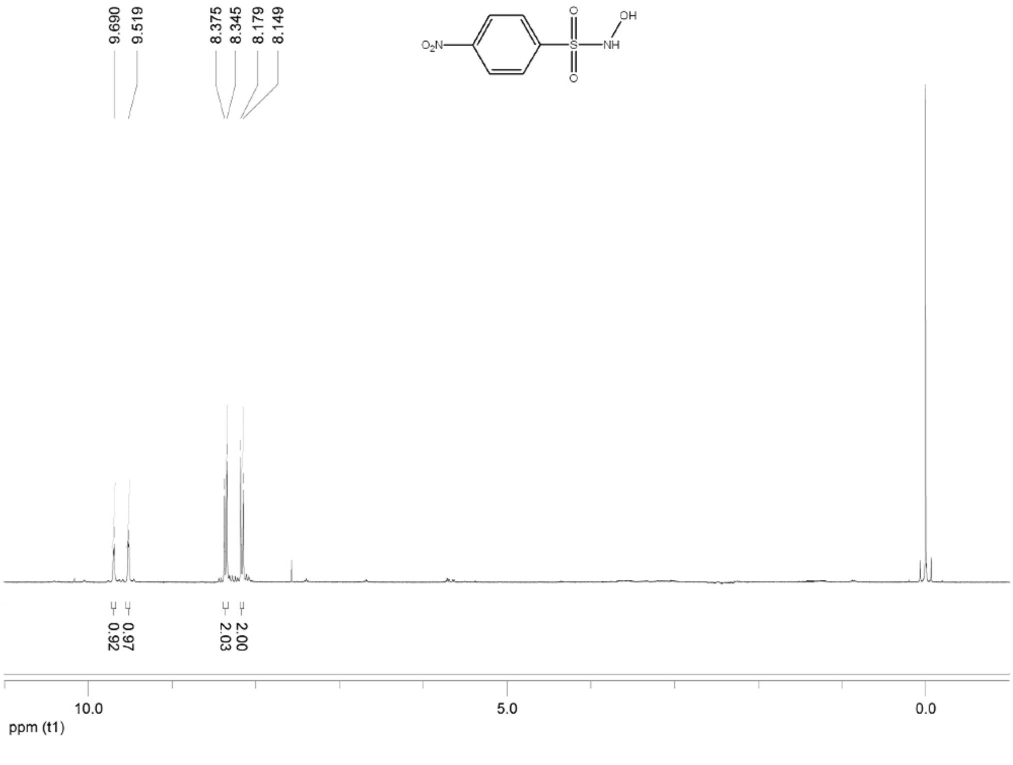

Figure S5. ^{1}H NMR of N-hydroxy-4-nitrobenzenesulfonamide.

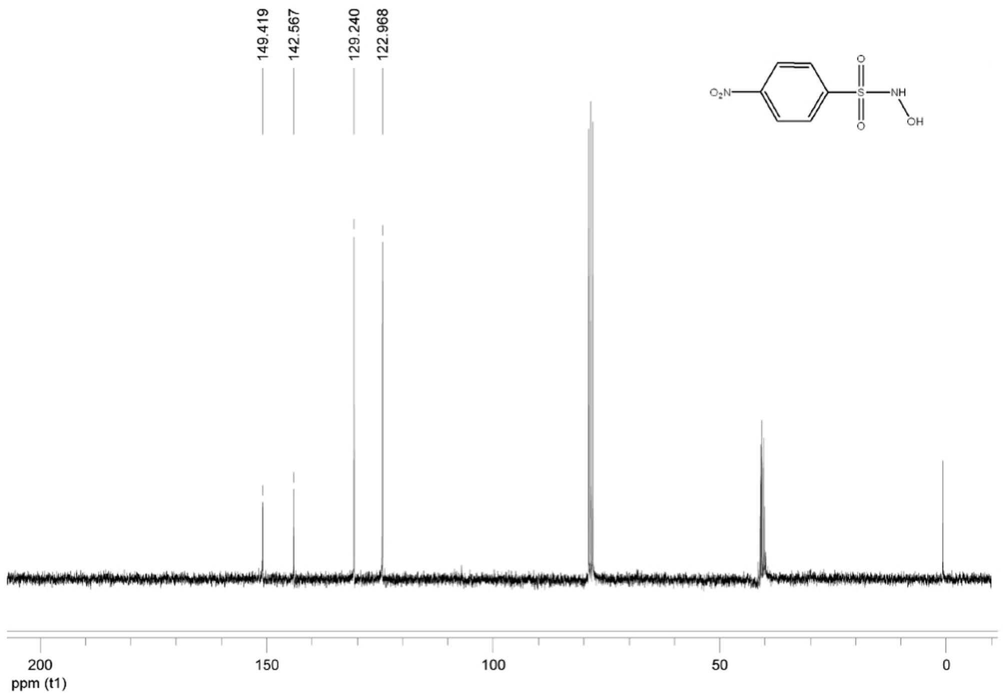

Figure S6. ^{13}C NMR of N-hydroxy-4-nitrobenzenesulfonamide.
